# Supplementary material for: Introducing MdTFL1 Promotes Heading Date and Produces Semi-Draft Phenotype in Rice
Source: Int J Mol Sci. 2023 Jun 20;24(12):10365. doi: 10.3390/ijms241210365 (PMC10299341; doi:10.3390/ijms241210365)
Supplement: Supplementary file 1 [file ijms-24-10365-s001.zip › ijms-2451313-supplementary.pdf]

International Journal of *Molecular Sciences*

MDPI

Supplementary Data

Article

Introducing *MdTFL1* Promotes Heading Date and Produces High-Throughput Phenotyping in Rice

Van Giap Do *et al.*

Supplementary Data

- Table S1. Sequences of primers used for gene cloning and genomic DNA PCR
- Table S2. Sequences of primers used for RT-PCR and qRT-PCR
- Figure S1. Genomic DNA PCR from rice callus
- Figure S2. Heading date of T0 (the year 2020) and T1 (the year 2021) plants
- Figure S3. Expression profile of endogenous-flowering-related genes in rice callus
- Figure S4. Phenotype and flowering time of T0 (the year 2020) and T1 (the year 2021) plants
- Figure S5. Phenotype of T2 plants (the year 2022)
- Figure S6. Expression profile of endogenous flowering-related gene and GA synthesis pathway genes in rice plants

Table S1. Sequences of primers used for gene cloning and genomic DNA PCR

Gene	Description		Primer sequence (5'-3')	Note
MdTFL1	TFL1-like protein	F	GGGGTACCATGAAAAGAGCCTCGGAG	Amplified the full length of target gene *MdTFL1*
		R	CGGGATCCCTAGCGTCTTCTAGCTG	
hptII	Hygromycin phos-photransferase	F	CTCGGAGGGCGAAGAATCTC	Amplified 563 bp of PCR product of *hptII* gene
		R	CAATGACCGCTGTTATGCGG	

Table S2. Sequences of primers used for RT-PCR and qRT-PCR

Gene	Description		Primer sequence (5'-3')	Accession No.
MdTFL1	TFL1-like protein	F	CTCGGAGGGCGAAGAATCTC	AB052994.1
		R	CAATGACCGCTGTTATGCGG	
OsUbi1	Polyubiquitin1	F	CACGGTTCAACAACATCCAG	NC_029261.1
		R	TGAAGACCCTGACTGGGAAG	
OsEHD1	EARLY HEADING DATE 1	F	TGGAGTATGGATCGCCCATT	NC_029265.1
		R	GCAGTTGCGGTTTCTAGGGT	
OsHD1	HEADING DATE 1	F	CACAATGCTGAGTGAGCAGC	NC_029261.1
		R	AGATGAACTCACGCTGTTGC	
OsHD3B	HEADING DATE 3B-like	F	GAATGGCCAGGTCGATTGGA	NC_029261.1
		R	CGCTTCCTACCAATTAGCGTG	
RFT	FLOWERING LOCUS T	F	CTGGTTTGCTTGGACTTGGC	NC_029264.1
		R	AGCTATGCCCTAGGTCCTCT	
OsGhd7	Transcription factor GHD7-like	F	TCCGTTCATGTCGATGGGAC	NC_029262.1
		R	CGGGAAGACGAAGGGGAATC	
OsMADS14	MADS-box transcription factor 14-like	F	AGCGCTCATAACGTTCAAGGA	NC_029258.1
		R	TGTGCATTTGTTGTTGAGATGGA	
OsMADS18	MADS-box transcription factor 18-like	F	CACTAACCGGGAGGAGCAAA	NC_029262.1
		R	GGTGAGTGCAGGGGCAAAAT	
OsMADS50	MADS-box transcription factor 50-like	F	TTATCCCAATTGGAGGGGCG	NC_029258.1
		R	ACCACCACTCCTTGCCTTTT	
Gn1a/ OsCKX2	Grain number 1a/ Cytokinin oxidase/dehydrogenase	F	GAGGTGTTCTACACGGTGGG	AB205193.1
		R	GCAGGTACTGCTTGTAGGCT	
OsEXPA3	Alpha-expansin-3	F	GGCAGAGCCTGTCCTTCATT	NM_001420295.1
		R	CAGAACTGCACCCAAGAGGT	
OsEXPA4	Expansin-A4-like	F	ATAGCACGGTGTCCTTCTTCG	NM_001420547.1
		R	AGCCCAAAACCTCTTCTCCAC	
OsGA20ox3	Gibberellin 20 oxidase 3	F	AGTATCGCACCGTCACGTTC	AK060318.1
		R	GCCGCTGTCAGGATCAGATT	
OsCPS2	ent-copalyl diphosphate synthase 2-like	F	CGGCAATGCTTCTAACAGCC	AB066271.1
		R	CGATTGTGCGAACTAGCAGC	
OsHD3A	HEADING DATE 3A-like	F	CTAGCGCACTTGTTTTCATGC	NC_029261.1
		R	GACGCTGCAGTAGTACCAGG	
OsKO1	ent-kaurene oxidase 1-like protein	F	CGGAAGAAGCCCCAATCCTT	NC_029261.1
		R	TTTATTGTCATGCAGCTGTTCC	
OsKO2	ent-kaurene oxidase 2-like protein	F	CTACAGAAGAAGCCCCGGTT	NC_029261.1
		R	TTCATTGTCATGCAGCTGTTCC	

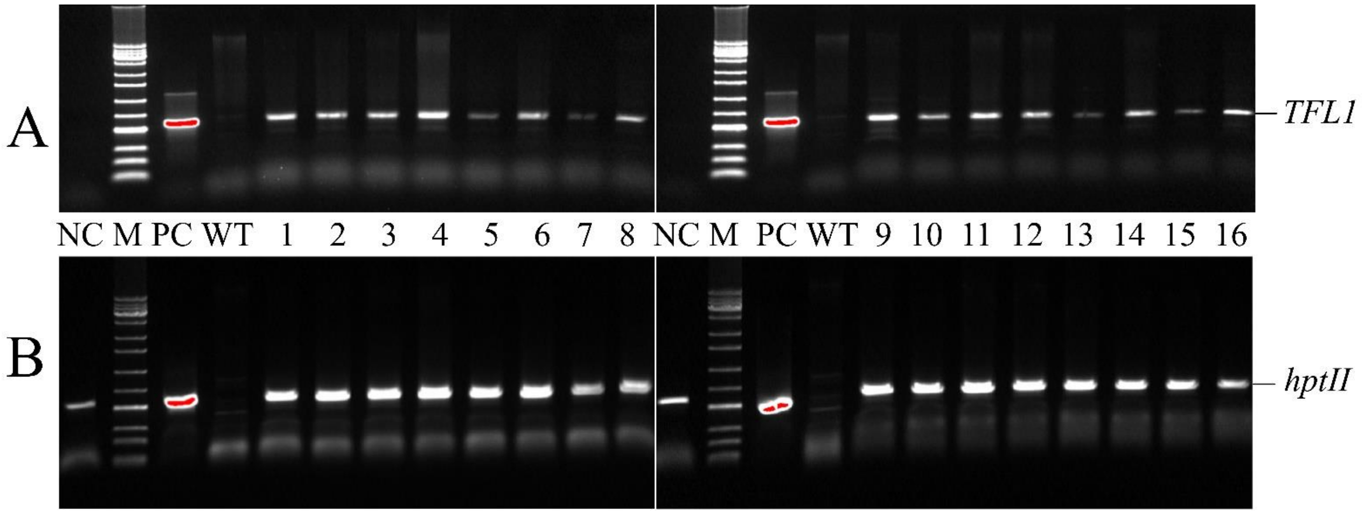

Figure S1. Genomic DNA PCR from rice callus. Detection of the target gene (*MdTFL1*) (**A**) and selection marker hygromycin phosphotransferase (*htpII*) (**B**) in transgenic rice callus. Lane PC, plasmid DNA of 3D::MdTFL1 used as positive controls; lane NC, genomic DNA of transgenic rice callus transformed with vector pMYD319 used as a mock; lane WT, genomic DNA of wild-type (non-transgenic callus) used as a negative control; lanes 1-16, independent 3D::MdTFL1 transgenic callus lines; lane M, 1 Kb plus DNA ladder marker.

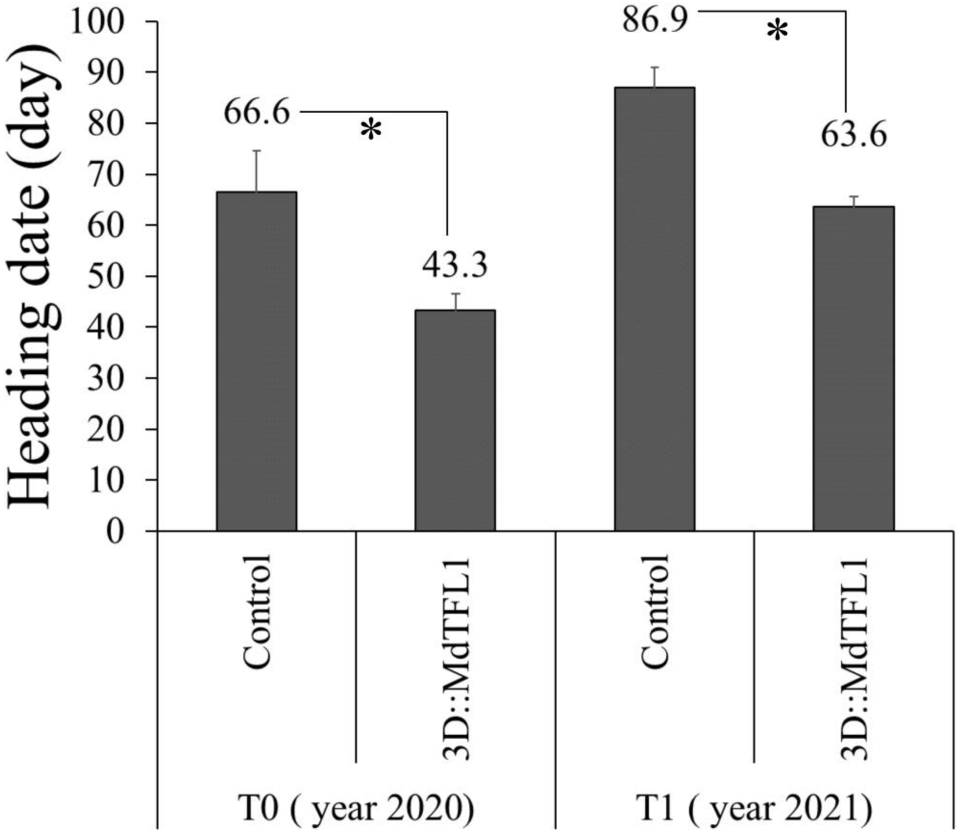

Figure S2. Heading date of T0 (the year 2020) and T1 (the year 2021) plants. The heading date of T0 plants was evaluated when the first panicle emerged after the in vitro seedlings were transferred to the soil. T1 plants were germinated from the seed of T0 plants, and their heading date was evaluated when the first panicle emerged after T1 seed sowing. Data are presented as mean ± SD ($n \geq 20$). The asterisks indicate significant differences compared to control (WT and mock) ($p < 0.05$).

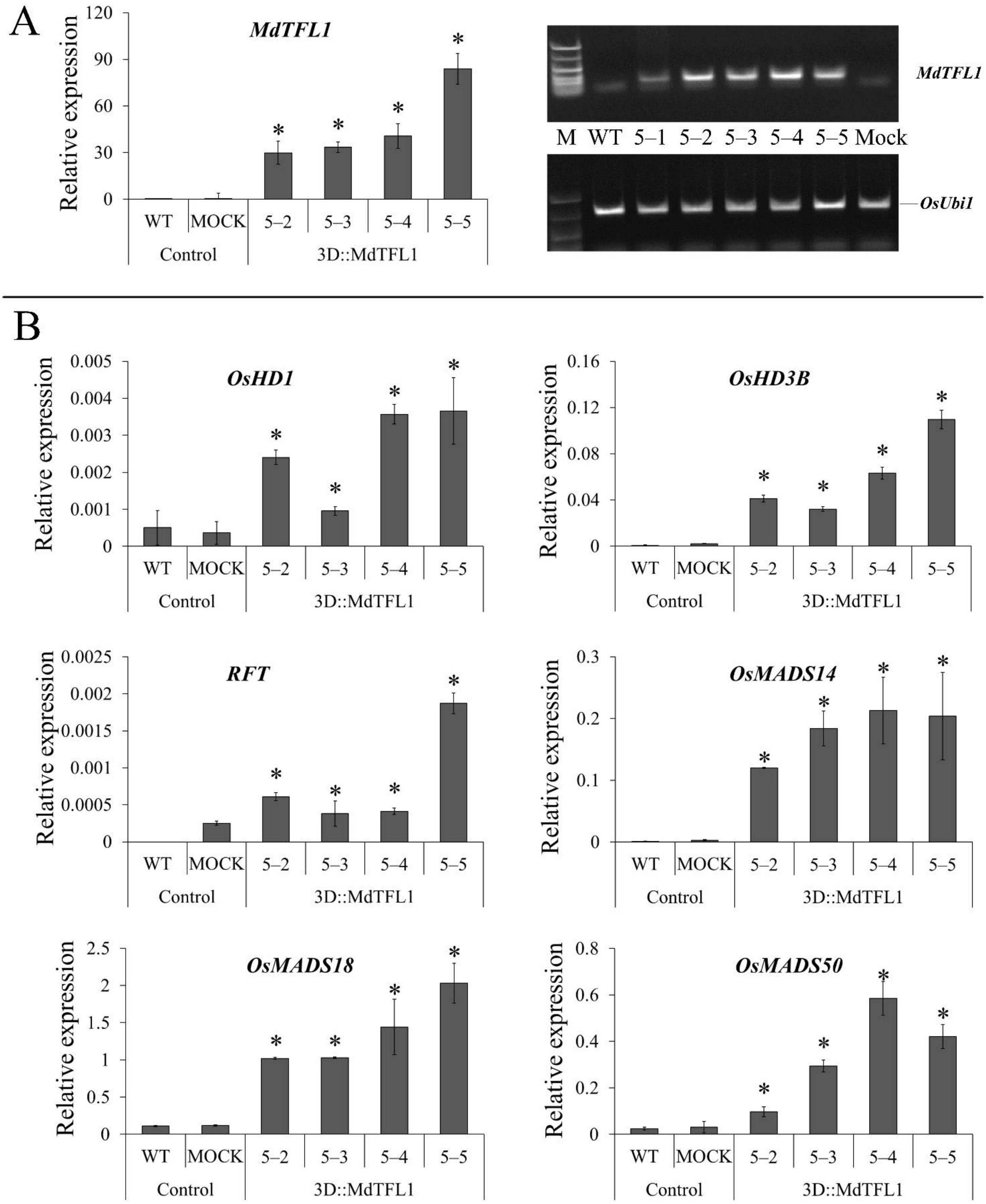

Figure S3. Expression profile of endogenous-flowering-related genes in rice callus. The expression level of *MdTFL1* (A) and rice flowering-related genes (B) was normalized to *OsUbi1*. Data represent the mean ± SD from three biological replicates. The asterisks indicate significant differences compared to control (WT and mock) ($p < 0.05$).

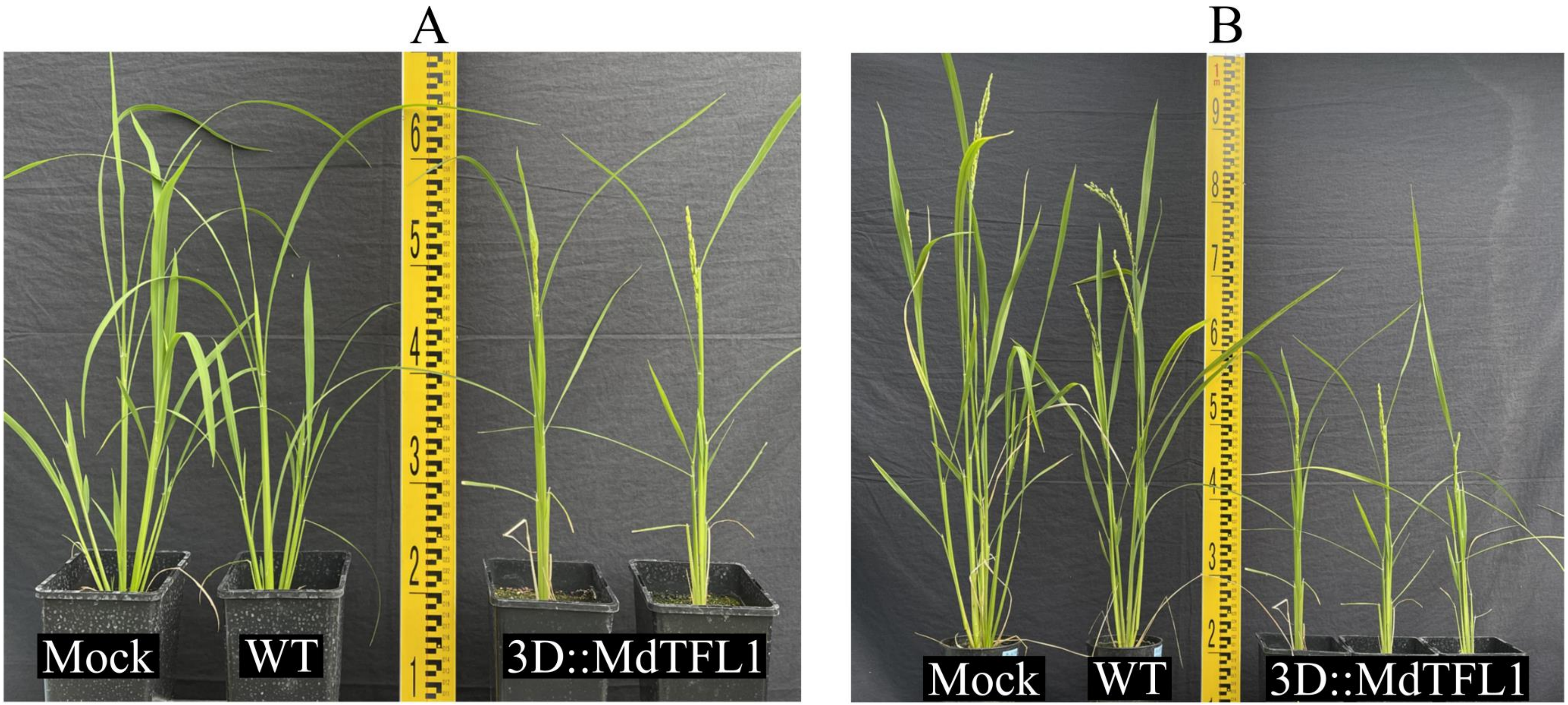

Figure S4. Phenotype of T0 plants (the year 2020) after transferring to the soil. (**A**) 3D::MdTFL1 transgenic rice plants showed early heading at 43 days after transferring the in vitro seedling to the soil while the control (mock and WT) plants remained at the vegetative stage. (**B)** Plant height of 3D::MdTFL1 transgenic rice plants was shorter than the control (mock and WT) plants at the same developmental stage (heading date).

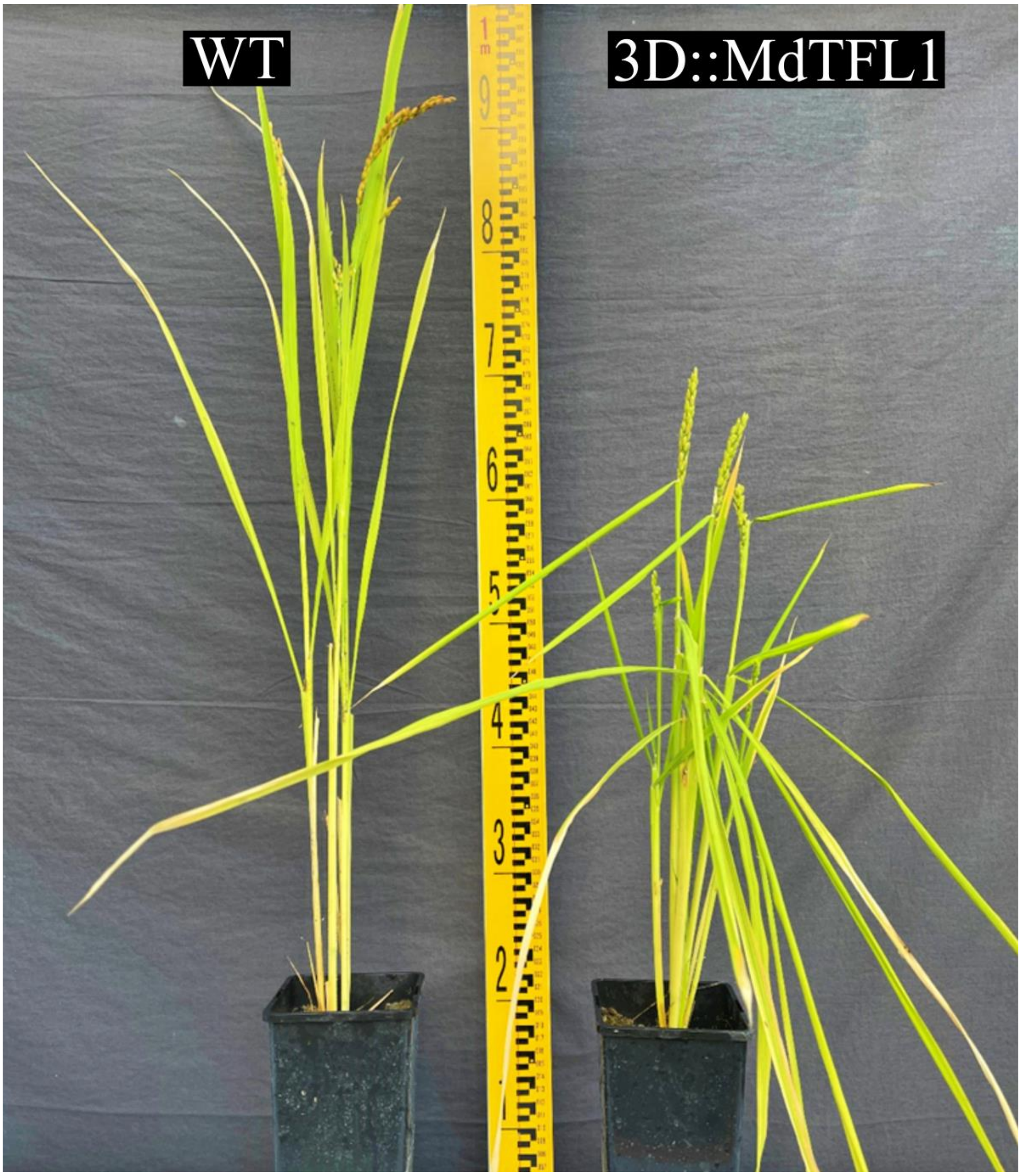

Figure S5. Phenotype of T2 plants (the year 2022). 3D::MdTFL1 transgenic rice plants showed a high-throughput phenotype that exhibited a semi-draft with inclined leaf angles.

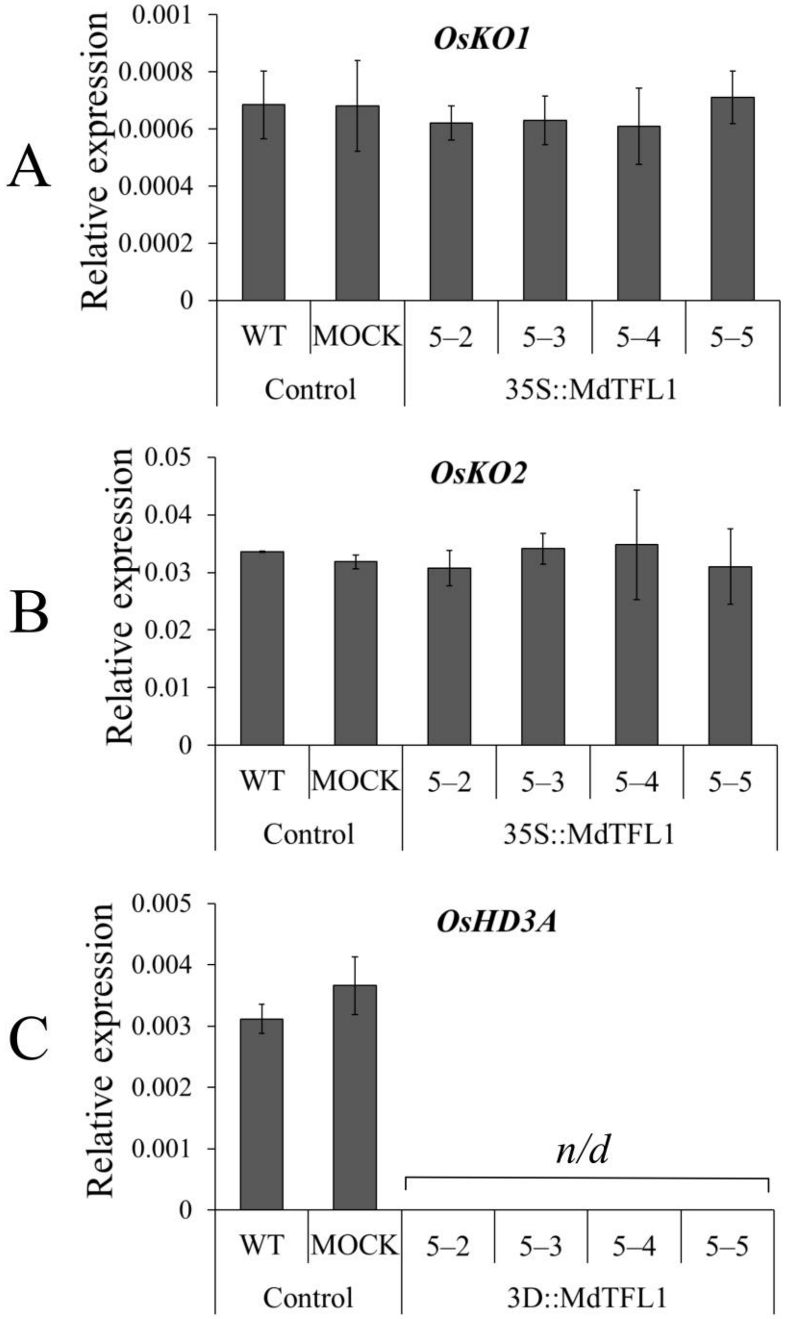

Figure S6. Expression profile of GA synthesis pathway and endogenous flowering-related genes and in rice plants. The expression level of *OsKO1* (A), *OsKO2* (B), and *OsHD3A* (A) was normalized to *OsUbi1*. Data represent the mean ± SD from three biological replicates. *n/d* indicates *OsHD3A* was not detected in 35S::MdTFL1.
